# Sexual vulnerability and HIV seroprevalence among the deaf and hearing impaired in Cameroon

**DOI:** 10.1186/1758-2652-13-5

**Published:** 2010-02-04

**Authors:** Adonis Touko, Célestin P Mboua, Peter M Tohmuntain, Anne B Perrot

**Affiliations:** 1 Cameroon Psychology Forum, Blv Rudolph Manga Bell, Immeuble Mony Onana, P.O. Box 8030 Yaounde, Cameroon; 2 Cameroon Psychology Forum and University of Yaounde 1, Blv Rudolph Manga Bell, Bd 2.002, P.O. Box 8030 Yaounde, Cameroon; 3 Department of Psychology, University of Yaounde 1, P.O. Box 755 Yaounde, Cameroon; 4 Care International in Cameroon, 1071, Avenue Winston Churchill, P.O. Box 422 Yaounde, Cameroon

## Abstract

**Background:**

This quantitative cross-sectional study examines sexual behaviour of a target group of hearing-impaired persons in Yaounde, the capital city of the Republic of Cameroon. It measures their HIV prevalence to enable assessment of their sexual vulnerability and to help reduce the gap in existing HIV serology data among people with disabilities in general and the deaf in particular.

**Methods:**

The snowball sampling procedure was adopted as an adequate approach to meet this hard-to-reach group. A total of 118 deaf participants were interviewed for the behavioural component, using sign language as a means of data collection, while 101 participants underwent HIV serology testing. Descriptive analyses were done for behavioural data with Epi info software, while sera were tested by health personnel, using rapid and confirmation test reagents.

**Results:**

From the results, it was clear that the hearing impaired were highly involved in risky sexual practices, as observed through major sexual indicators, such as: age at first sexual intercourse; condom use; and knowledge of sexually transmitted infections and AIDS. Furthermore, it was noted that the HIV prevalence rate of the hearing impaired in the capital of Cameroon was 4%, close to the prevalence in the city's general population (4.7%).

**Conclusions:**

Such results suggest that there is a need for in-depth behavioural research and serological studies in this domain to better understand the determinants of risky sexual behaviour among the hearing impaired, and to propose operational prevention approaches for this group.

## Background

The fight against HIV/AIDS has shown that the exclusion of people living with disabilities (PWD) is an influential vulnerability factor that may slow down prevention measures. Stigmatization and discrimination, generally associated with living with a disability, constitutes an important sexual vulnerability factor that adds to other inherent bio-psychosocial factors of disability. Research has constantly proved that PWD are victims of exclusion in the management of the health crisis generated by the AIDS pandemic [1-5].

As indicated by a number of studies, most people assume that PWD are asexual, or less sexually active, and that they are less exposed to such sexual risks as sexually transmitted infections (STIs) and HIV [6,7]. On the same note, many non-governmental organization officials, programme directors and other influential people at the operational level rarely design projects with the thought of positioning PWD at the centre of their interventions [3,8]. Furthermore, there is very little evidence of the engagement of international donors in programmes that target PWD [9]. Thus, while considerable efforts have been made to reduce the dramatic and disabling effects of HIV/AIDS in the general population, there have been very few measures to build intervention programmes that minimize the impact of this disease on people with disabilities.

Currently, while official discourse on the universal character of the fight against the AIDS pandemic is becoming more persistent, there exist, paradoxically, very few prevention, care support and HIV treatment initiatives targeting PWD. Yet this social minority constitutes 10% of the world's population, according to World Health Organization and World Bank estimates [10,11]. This represents more than 400 million people living with a physical, sensory, intellectual or mental health disability in the developing world [12].

Sadly, the situation is more dramatic in women with disabilities, who are also victims of social inequalities [13] and violence [14,15] as far as sexual practices are concerned. Marginalization of PWD is conspicuous in the domain of research with limited information available, as observed through a literature review, and with scanty scientific exploration of the relationship between AIDS and disability [16]. One of the first large-scale studies to highlight this situation was conducted about five years ago within the framework of the World Bank's Global HIV/AIDS Program of Action [17]. The results confirmed that PWD have been excluded from efforts in the fight against AIDS, although this disease represents a serious threat to them. In this light, it is clear that this pandemic cannot be overcome without systematic involvement of PWD.

It should also be noted that the few efforts that have been undertaken towards the integration of disability as a vulnerability factor in the fight against HIV/AIDS are unequally dispersed in different parts of the world. In contrast to the experience in many countries in North and South America (especially the USA and Canada) and in Europe, many developing countries are yet to include disability in their HIV/AIDS programming initiatives, as noted by the Kampala Declaration [9]. This is particularly the case in Africa, where there is a high degree of stigmatization and discrimination, although South Africa seems to be playing a leading role in this regard [18].

A thorough review of literature on disability and HIV/AIDS in Africa indicated that the only African countries in which at least three studies on HIV and disability have been conducted are: South Africa (13 cases of research work); Zimbabwe (six cases); Kenya (four cases); Uganda (three cases); and Nigeria (three cases) [19]. No country from west and central Africa is mentioned, suggesting that the information gap is more critical there. Lack of data darkens an already blurred area.

To counteract this situation, stakeholders and some authors have recommended that research, particularly, should address these appalling gaps and so capture the attention of decision makers [9,18]. However, very little attention has been given to research, especially in gathering prevalence data, which would describe epidemic trends and orient intervention measures [8,20]. The present study is a response to this situation. It intends to evaluate sexual risk factors and determine HIV prevalence among the young deaf people of Yaounde in Cameroon as a means of reducing the deficiency of information on behavioural and HIV serology indicators among this social minority in Cameroon in particular and Africa in general.

## Methods

### Study type and sampling procedures

This was a quantitative cross-sectional study, combined with a serology component, with the aim of better understanding sexual behaviour and determining HIV seroprevalence within the deaf in Yaounde, the capital of Cameroon [21]. Respondents were identified in the five subdivisions of Yaounde through application of the "snowball" sampling procedure [22]. This procedure is often used for studies on hard-to-reach groups and hidden groups, such as men who have sex with men [23].

For the behavioural survey, recruitment in each of the subdivisions was done with the assistance of leaders of deaf and hearing impaired associations. The interview process followed a progressive path. First, interviews were conducted with leaders of associations, hearing-impaired persons known by leaders of associations, and hearing-impaired persons known by the persons recruited. Second, each informant was used as a resource person for the identification of one or more other deaf people in the same subdivision until a total of 25 informants per subdivision had been identified. These people were then interviewed.

The necessity of using this mode of recruitment is largely explained by the distribution of the deaf and hearing impaired in the city of Yaounde. As is the case in most African urban areas, a minority of deaf and hearing impaired are affiliated to association networks, whereas the vast majority can be met only at their homes, generally situated in poverty-stricken quarters, or at their work places. Interviews were conducted using French Sign Language (FSL) at settlements where deaf respondents were found.

The serology survey was organized in close collaboration with local Ministry of Health services, which brought in the necessary personnel to manipulate the material acquired for this operation. At the end of the interviews, each interviewee was given the option of having a free HIV screening test at one of the nearest health centres, where arrangements had previously been made for these tests. Volunteers were given special cards to go to these health centres, and reticent participants were told that they were free to have these tests if they changed their minds. At each health centre, a team made up of a psychologist, a health practitioner and a FSL instructor carried out pre-test counselling sessions and screening tests for a period of one month.

### Construction and administration of tools

For the purpose of this quantitative cross-sectional study, an anonymous pre-coded questionnaire was constructed following conventional indicators of knowledge, attitudes and practices on sexuality and HIV/AIDS. The indicators explored were essentially related to the following themes: initiation into sexuality; sexual behaviour; condom use; sexually transmitted infections; and HIV/AIDS. Questionnaires were administered by both male and female interviewers, selected from those young deaf and hearing-impaired people who showed proof of good mastery of sign language.

In order to build the abilities of the selected interviewers to manipulate the data collection tool, FSL instructors were trained first on the techniques of social data collection, enabling them to train their young deaf peers who would be interviewers. To evaluate the abilities of deaf peers in the data collection process, a pre-survey was conducted as a test through which FSL teachers and researchers verified the deaf interviewers' mastery of the data collection tool. This pre-survey highlighted the need to adjust the instrument to meet the modest understanding of the targeted population that was mostly made up of people with a very low literacy level.

Other important concerns were pointed out during the study preparation process and during the administration of the questionnaire. Among these issues was the necessity to ask each question several times to be sure it had been well understood by the deaf participant. Furthermore, considering that confidentiality is a prerequisite condition for ethical social research, the adoption of sign language as a coded mode of expression quickly created favourable conditions to ensure confidentiality: the hearing people who were present could not easily understand the conversation between the interviewer and the deaf interviewee.

Interviews were conducted wherever the respondents were met: in households, workplaces and training centres. Overall, no major problem was encountered between the deaf and the interviewers during the interview process.

A total of 101 interviewees (56% males and 44% females) voluntarily underwent HIV screening tests. Sera were collected in test tubes and tests were performed in a laboratory, using Determine HIV-1/2 (Inverness Medical Innovations Inc., Waltham, MA, USA) rapid reagents. Positive and undetermined results were confirmed with ImmunoComb II HIV 1&2 BiSpot (Orgenics LTD, Yavne, Israel). All the candidates were invited for post-test counselling sessions, and those who tested positive were referred to the nearest care provision unit to benefit from follow-up services, as prescribed by the national care support algorithm [24,25].

### Ethical concerns

Ethical issues were considered for both the behavioural and serology component of the study. Before administration of the questionnaires, clear explanations were given to all participants about the purpose of the study and the sampling procedure. Interviewers were advised to look for an appropriate venue that was likely to ensure confidentiality, and to obtain the consent of each informant before commencing the interview. For legal minors (age below 18), consents were sought from the guardians or parents, depending on the interview setting.

At the health institutions, participants were invited to give their informed consent by signing a form after carefully reading its contents and having all their questions satisfactorily answered. The codification procedure that was adopted to avoid information leakage, as well as to ensure other confidentiality aspects of the operation, was explained in simple language to each of the interviewees before beginning collection of blood. Post-counselling sessions were planned two days after the screening test, and results were delivered only to the concerned individuals or legal guardians.

### Data analysis

Data were entered and analysed using Epi Info software. Statistical analyses were essentially descriptive, with proportions as a first-level comparison. Whenever necessary, the Chi2 test was performed to verify independence or association between variables. Results were presented using sex as main reference to highlight any eventual gender effect.

## Results

### Sample characteristics

The sample showed a strong domination of unmarried people (77%), followed by those in cohabitation (20%); only 1.6% were married people. Regarding education, 97% declared that they had been to school, but this was limited to primary school level for the majority of cases (60%). Of those at the post-primary school level, none had reached high school. Regarding occupational status, respondents were grouped into three main categories: pupils/students (30%); those doing any given type of job (47%); and those with no occupation (23%). It is worth noting that persons who were working were engaged mainly in casual work, such as apprenticeships. There was a relatively clear social division of labour between males (apprentice builders, carpenters, car washers and shoe menders) and females (hair dressers, apprentice seamstresses and vegetable vendors).

For the behavioural component of the study, a total of 118 interview forms out of 125 were validated, representing 94.4% of all respondents. Questionnaires with significant loopholes or gaps were eliminated. The sample was made up of 50.8% males and 49.2% females. Ages varied between 15 and 34 years, with an average age of 24.5 years for males and 22.3 years for females. Most of the respondents were Christians (86.5%); others were Muslims (4.2%), followed by traditional African religions (6.7%) and no religion (2.5%).

### Sexual behaviour and risk taking

In all, 80% of respondents had had sexual intercourse. No difference between men and women was observed on that matter. The average age of first sexual intercourse was 17 years for men and 16 years for women, with 25% of all the respondents, men and women, reporting having had this experience before the age of 15 years. The average age for the first experience in cohabitation was 24 years for men and 22 years for women.

Among the sexually active interviewees, men (53%) and women (54.3%) were engaged in multiple concurrent sexual relationships in the 12 months prior to the study for regular partners, with no gender difference. Most of those interviewees who had had more than two partners were those not cohabitating (51.4%), as against those cohabitating (29.6%), for the considered period (chi2 = 3.74, p = 0.05). For casual partners, multi-partner sexual relationships were higher for men (42.6%) than women (13.5%) (chi2 = 8.35, p = 0.003). Commercial sex workers ranked highest in casual sexual relations, making up 44% of sexually active women and 64% of men, during the 12 months prior to this study. Similarly, the deaf who did casual work were more engaged in commercial sex (55.1%) than pupils/students (18.4%) and the "unemployed" (26.5%), either in buying (men) or selling (women).

### Condom use

Although there was a marked increase in the use of condoms (85%), it was noticed that a substantial portion (15%) of the sexually active had never used a condom in their lifetimes; these people were more likely to be females (80%) than males (20%) (chi2 = 7.9, p = 0.007). Careful consideration of data related to each individual's most recent casual sexual intercourse highlights a critical situation: 53% of deaf respondents did not use a condom during this sexual act. Main reasons for not using a condom during this act were: confidence in partner (55%); refusal by partner (20%); and unavailability of a condom (10%).

For respondents who used a condom during their most recent sexual act, its use was motivated mainly by the need to: prevent HIV/AIDS (54%); prevent a pregnancy (28%); and prevent an STI (10.6%). Analysis showed that in the majority of cases (60%), the interviewee proposed condom use during the most recent sexual act. However, there was a striking difference between the proportion of women who took such an initiative (29.8%) compared with men (70.2%) (chi2 = 5, p = 0.025). Consistent arguments for the timidity and the incapacity of the deaf towards condom use were summarized in three major points: immediate rejection of condom; non-mastery of the use of condom; and inability to put up an argument and convince a partner on the proposal.

### Knowledge, attitudes and practices related to HIV/AIDS

With regards to HIV/AIDS in particular, very few deaf considered themselves as potentially at risk of contracting HIV. Only 36% of the respondents believed that they could easily contract the virus. This indicator was explained by the weak self-perception of being at risk, especially by women (30%), as compared with men (41%) (chi2 = 1.63, p = 0.02).

The weak self-perception of being at risk can be largely attributed to several false beliefs on the part of the deaf concerning the disease. Such beliefs include the following: 30.2% believed that AIDS could be cured; 42.1% assumed that it was possible to identify an HIV carrier from his or her physical appearance; and 44.2% were of the opinion that someone who was apparently in good health could not transmit the HIV.

### HIV prevalence

Initially, 73% of participants declared their willingness to undergo HIV screening tests; the remainder expressed fears about the tests. Finally, 85% of participants agreed to undergo the HIV screening tests following some contextual encouraging measures, such as, sending deaf peers to explain the importance of early detection and early care support.

Results indicated a global seroprevalence rate of 4%, with a remarkable sex difference: a total of three females and only one male. Although some of those found to be HIV positive understood the high risk of HIV transmission due to their sexual behaviour, they had not used condoms during their most recent casual sexual encounter. All the HIV-positive cases belonged to the 20-34 year age group, were unmarried, and were engaged in casual work.

## Discussion

### Methodological aspects

Surveys of hard-to-reach populations imply some specific elaborations on the methodology procedure to be applied in order to obtain a consistent sample collected in accordance with scientifically prescribed norms. Sampling procedures in social studies are usually non-probabilistic where a statistical data base does not exist. In Cameroon, the available data on census [26,21] did not consider disability as a study variable, leading to methodological difficulties in determining a random sample in studies that target PWD.

Determining a random sample becomes more complex when applied to hard-to-reach populations, such as the deaf and the hearing impaired that cannot be easily identified, and more so, when involved with the specificity of communicating with them. Thus, working with such groups is fraught with technical and procedural difficulties for which research has proposed several types of solutions. In this perspective, pluralistic approaches have been recommended, using diverse terminologies to identify potential participants, such as respondent-driven sampling, time-space sampling [27] or snowball sampling [22]. In its own manner, each of these methods deals with the problem of the absence of a sampling base. Each researcher is advised to adopt the approach that is best suited for his/her work.

With regard to the conditions in which interviews are carried out, the diversity of targeted groups produces a diversity of problems to be resolved, and this implies that the researcher should adopt an innovative and flexible spirit that is appropriate to each situation. Concerning the deaf and the hearing impaired, communication difficulty constitutes a major challenge. To fill this communication gap within the framework of this study, FSL instructors were trained in the techniques of social data collection. They were then employed as intermediaries to train the deaf and hearing-impaired interviewers who were finally responsible for the collection of data among their peers.

Using peers as a means to reach the sample, or tracing the target, has a two-way effect. It carries the risk of reducing confidentiality given that these are people who may know each other. However, it also constitutes an effective way of implicating the target group in the process. The latter falls in line with this widely shared maxim among PWD: "Nothing about us, without us" [28,29]. Furthermore, the implication of the deaf as facilitators with their own peers was a crucial factor in social mobilization and in establishing confident relationships relating to the survey with this group, as indicated in some programmes [29,30].

### Social vulnerability

The target population presented extreme social vulnerability. The first feature to be noted was the low literacy level. While the majority of respondents were literate, it is worthwhile reiterating that their level of education was very modest. This is easily seen at the level of social status and in the distribution of social roles of those employed. Very few of them did anything apart from manual labour. For this reason, most of them were poor, living in shanty towns and filthy quarters, thereby becoming prey to absolute vulnerability on a hygienic and socio-educative plane.

As indicated by the United Nations Development Programme, the level of education for disabled persons is generally low, at 3%, with a significant gender inequality: for women, this level is just 1% [31]. Recent studies conducted by the United Nations Educational, Scientific and Cultural Organization give a more dramatic picture of education of children with disability: only 1% to 2% have access to basic education [32]. This low level of education among PWD, with women bearing the brunt because of gender-related inequalities, has been reported elsewhere. Uganda can be considered as an illustrative example, with 49% of women with disability and 88% of men with disability having been to school [33]. As reflected by this low level of education, occupational activities by the interviewed deaf were limited mostly to casual work.

### Sexual risk behaviour

Many types of risky sexual behaviour were reported during this study. These included unprotected sex and multiple concurrent sexual relationships. Risky sexual behavioural patterns are repeatedly reported in studies related to PWD in general and to the hearing impaired in particular. Such behaviour highlights the vulnerability factors that can explain the high prevalence found in this population in Yaounde. In this case, risky sexual relations can be explained in a dual context: (1) factors engendered by society; and (2) individual factors related to PWD.

With regard to sexual risk behaviour and practices engendered by society, several examples were found in the literature review, notably concerning rape and sexual abuse [33,14]. In an article dedicated to this theme, Groce [6] presents rape of PWD as acts motivated by several factors, such as belief in the virginity of the disabled and the fantasy of "purification through virgins". Meanwhile, Hanass-Hancock talks of "sexual purification rituals" to describe the same scenario in South African populations [18]. Thus, in most cultural contexts, acts of rape on PWD are essentially motivated by socio-cultural beliefs and prejudices.

In this perspective, these analysts agree that in many cases, seropositive persons who perpetrate rape believe that by having sexual intercourse with PWD, the virus will be purged from their systems [6]. Such false beliefs, coupled with the impairment of PWD, are at the heart of most of the sexual abuses they suffer, and definitely expose them to STIs and HIV. As a conclusion to these issues, research has brought evidence that, in reality, the incidence of perpetration of acts of sexual violence are significantly higher towards the deaf and the hearing impaired than the general population [34,35].

At the individual level, the multiplicity of PWD's sexual partners corresponds to a self-validation fantasy that leads to the continuing search for a positive self-image through acts of seduction. In this perspective, the deaf and the hearing impaired believe that having several sexual partners provides opportunities to show their attractive abilities to their peers, or to demonstrate that their sexual prowess is at least equal to that of others. It can also be considered as a tentative attempt to counteract their sexual marginalization and inferiority complex. This process of filling a symbolic gap has been analyzed by well-known psychologists. For instance, Freud refers to it as a "castration complex" [36], and Adler treats it as a consecutive compensation of an inferiority complex [37]. In a more practical description, Bernard presented the case of a young deaf boy, who had an unequivocal determination to find a homosexual sex partner among hearing people [38].

Our research showed that in the 12 months preceding the survey, multiple concurrent sexual relationships among non-cohabiting partners affected 43% of men and 13% of women, as against 41% of men and 10% of women in the general population of the same age group, as indicated in the last Cameroon Demographic and Health Survey [39]. From such findings, deaf persons may be more prone to the practice of multi-partner sexual relationships than the general population.

However, this situation is still debatable: some sources indicate that differences in multi-partner sexual relationships between hearing people and the hearing impaired are statistically insignificant [35]. For this reason, it is necessary to conduct studies on this aspect, focusing on both multi-partnerships and sexual orientation. This is particularly important given that multiple concurrent sexual relationships within people of the same subgroup may favour the rapid spread of STIs within this subpopulation.

### Occurrence of STIs

Knowledge of STIs was very limited among the target (50%), representing another sexual vulnerability factor faced by PWD compared with the general population. Indeed, several studies carried out in recent years in Cameroon indicate a higher level of awareness in both the general population [40] and specific groups [41]. This said, more than mere knowledge of these infections, the incidence of STI episodes showed that deaf people were three times more likely to be infected than their counterparts in the general population: 30% of males and 10% of females reported having had an STI episode compared with only 6.4% of males and 4.5% of females in the same age group in the general population [39].

### Seroprevalence

Data related to HIV seroprevalence in the deaf population contradicts the social prejudice that these people are sexually inactive. The level of prevalence in this target population (4%) is fairly close to the average of the city in which the research was conducted (4.7%) and to the national average (5.5%) [39]. This is an indication of the risky behaviour in which this target group is involved.

To date, very few studies have been carried out to address the problem of HIV seroprevalence among PWD. There is, therefore, a crucial gap as far as data and information are concerned to better understand the situation and elaborate on its patterns. Prevalence trends may be at least equal to, or higher, in this subgroup than in the general population. For example, basing their arguments on demographic data and seroprevalence, as indicated by the Demographic and Health Survey, [42] the Kenya Disability Association estimates seroprevalence among populations of PWD in that country at 10%, against 7% at the national level [43]. Such a situation may not be specific to Kenya: similar figures have recently been reported in another African country, Rwanda [44], and earlier in the US [45]. From these trends, it appears that there is an absolute urgency to intensify prevention and treatment actions among this social minority.

## Conclusions

This study clearly highlights hearing impairment as an important risk factor - both a psychological and social vulnerability factor - for contracting STIs and HIV in Cameroon. In fact, there is converging information that the general population's social perception of the hard of hearing, as well as their own self-image, constitute elements that weaken the ability of the deaf and the hearing impaired in making safe sexual choices, thereby further exposing them to sexual risks. Sexual activity is real and intense among our sample, who are, in most cases, more exposed to sexual risk than hearing persons, contrary to social clichés, which assume that these people are asexual.

To tackle this vulnerability, it is crucial to intensify research efforts to increase the knowledge of the impact of HIV/AIDS among PWD in general and the hearing impaired in particular. This will greatly contribute to furnishing decision makers and programme managers with adequate and required information, encouraging them to initiate education and HIV prevention projects. Finally, the research results will be used as an advocacy tool for the promotion of an inclusive and participatory approach in the fight against AIDS, with a well-defined objective towards scaling down the progression of the virus among hearing-impaired persons.

## Competing interests

The authors declare that they have no competing interests.

## Authors' contributions

AT assembled all the necessary data and wrote the original French version of the article. MCP participated in the early analysis, did insight reading of the original French version of the article and made some critical adjustments. PT assisted in data analysis, translated the original French version of the article into English, participated in the English manuscript revision and also carried out the final English arrangements. AP proposed the dissemination of the information through a scientific article, assisted in data analysis, participated in the revision of the final manuscript and provided some useful comments. All authors read and gave their approval for the final manuscript.
